# SHOX2 cooperates with STAT3 to promote breast cancer metastasis through the transcriptional activation of WASF3

**DOI:** 10.1186/s13046-021-02083-6

**Published:** 2021-08-31

**Authors:** Yong Teng, Reid Loveless, Elayne M Benson, Li Sun, Austin Y Shull, Chloe Shay

**Affiliations:** 1 Department of Hematology and Medical Oncology, Winship Cancer Institute, Emory University School of Medicine, 201 Dowman Dr, GA 30322 Atlanta, USA; 2 Department of Oral Biology and Diagnostic Sciences, Georgia Cancer Center, Augusta University, 30912 Augusta, GA USA; 3 Department of Biology, Presbyterian College, 29325 Clinton, SC USA; 4 Emory Children’s Center, Emory University School of Medicine, 30322 Atlanta, GA USA

**Keywords:** SHOX2, STAT3, WASF3, Metastasis, Transcriptional activation, Breast cancer

## Abstract

**Background:**

Metastasis is most often the root cause of cancer-related death. Human short stature homeobox 2 (SHOX2), a homeodomain transcription factor, is a novel inducer of epithelial-to-mesenchymal transition in breast cancer cells, though its exact role and underlying mechanisms in metastasis are not well understood.

**Methods:**

TCGA analysis was performed to identify the clinical relevance of SHOX2 in breast cancer. Gene depletion was achieved by short hairpin RNA and small interfering RNA. Molecular regulations and alterations were assessed by Western blotting, immunoprecipitation, immunohistochemistry, qRT-PCR, chromatin immunoprecipitation coupled with qPCR (ChIP-qPCR), and ChIP/re-ChIP. The impact of SHOX2 signaling on tumor growth and metastasis was evaluated in orthotopic breast tumor mice.

**Results:**

The expression level of SHOX2 is strongly associated with poor distant metastasis-free survival in breast cancer patients and inactivation of SHOX2 suppresses breast tumor growth and metastasis in mice. In breast cancer cells, SHOX2 directly activates Wiskott-Aldridge syndrome protein family member 3 (WASF3), a metastasis-promoting gene, at the transcriptional level, leading to a significant increase in metastatic potential. Mechanistically, SHOX2 activates signal transducer and activator of transcription 3 (STAT3) and recruits it to the WASF3 promoter, where STAT3 cooperates with SHOX2 to form a functional immunocomplex to promote WASF3 transcriptional activity in breast cancer cells. WASF3 knockdown abrogates SHOX2-induced metastasis, but not SHOX2-dependent tumorigenesis.

**Conclusions:**

These findings provide a critical link between the SHOX2-STAT3-WASF3 signaling axis and metastasis and suggest that the targeting of this signaling node may represent a valuable alternative strategy for combating breast cancer metastasis.

**Supplementary Information:**

The online version contains supplementary material available at 10.1186/s13046-021-02083-6.

## Background

Human short stature homeobox 2 (SHOX2), also known as OG12, OG12X, or SHOT, and its murine counterpart share 99 % sequence identity at the amino acid level and exhibit similar expression patterns during embryonic development [1, 2]. During the early stages of differentiation, heterologous expression of SHOX2 in mouse embryonic stem cells strongly favors the genetic programs for pacemaker development, leading to an increase in automaticity in vitro and the induction of biological pacing upon transplantation in vivo [3]. In contrast, SHOX2 inactivation in mice significantly reduces bone formation in the hard palate and results in an anterior clefting phenotype [4]. In particular, SHOX2 controls osteogenic differentiation and pattern formation during hard palate development through its regulation of pattern specification and skeletogenic genes that are associated with accessible chromatin in the anterior palate [5].

It has been demonstrated that the SHOX2 locus is frequently amplified and hypermethylated in lung cancers [6]. High SHOX2 copy number variation in cancer cells leads to elevated levels of extracellular methylated SHOX2 DNA that can be used for diagnostic and prognostic purposes for patients with advanced lung cancer. Interestingly, one recent study reported that SHOX2 was among the most up-regulated genes following the induction of STAT3 activation in *Helicobacter pylori*-infected patients with gastric cancer [7], which suggests that SHOX2 may contribute to the initiation and progression of gastric carcinogenesis. Our previous studies showed that high expression levels of SHOX2 are significantly associated with mesenchymal-like cell shape and poor survival of breast cancer patients [8]. A systematic evaluation of miRNAs further revealed that SHOX2 expression levels negatively correlate with the expression levels of miR-375, a well-known microRNA involved in the migration and invasion of breast cancer cells [9]. As a direct miR-375 target, SHOX2 promotes the epithelial-to-mesenchymal transition (EMT) of breast cancer cells through the up-regulation of transforming growth factor β (TNF-β) signaling [8]. The mechanisms by which SHOX2 contributes to the development and progression of breast cancer, however, remain largely unknown.

Here, we show for the first time that SHOX2 drives breast cancer metastasis through the regulation of Wiskott-Aldridge Syndrome (WAS) protein family member 3 (WASF3), an actin cytoskeleton controlling and metastasis-promoting gene [10–12]. We observed that SHOX2 activates signal transducer and activator of transcription 3 (STAT3) and it is required for STAT3 recruitment to the WASF3 promoter. We further demonstrated that STAT3 cooperates with SHOX2 to form a functional immunocomplex on the WASF3 promoter to promote WASF3 transcriptional activity in breast cancer cells. These findings improve our mechanistic understanding of this metastatic disease and may translate into a novel strategy to interrupt the SHOX2-STAT3-WASF3 signaling axis within the metastatic cascade for therapeutic purposes.

## Materials and methods

### Cell lines and standard assays

The breast cancer cell lines MDA-MB-231 and T47D were obtained from the American Type Culture Collection (ATCC, Rockville, MD) and have been verified using SNP-CGH for characteristic cytogenetic changes. Lentiviral transduction, transient transfections, cell proliferation assays, Transwell invasion assays, luciferase reporter assays, Western blotting, and QRT-PCR analysis were carried out as described previously [8, 13–15].

### Constructs, antibodies, and reagents

siRNA against WASF3 and the pLKO lentiviral vectors containing an shRNA against SHOX2 or STAT3 were purchased from Open Biosystems (Huntsville, AL). Coding sequences of human HA-tagged WASF3 and FLAG-tagged SHOX2 were respectively cloned into pCDH-CMV-MCS-EF1 lentivector (System Biosciences, Mountain View, CA) as described previously [8, 13]. The pGL-WASF3 promoter constructs (-350/+494, -650/+494, -1250/+494) were generated as described previously [16]. The following primary antibodies were used in Western blot assays: anti-WASF3, anti-STAT3, anti-pSTAT3 (Tyr 705), and anti-E-cadherin antibodies (Cell Signaling Technology, MA); anti-HA, anti-FLAG, and anti-β-Actin antibodies (Sigma-Aldrich, MO); and anti-SHOX2 antibody (Abcam, MA). The STAT3 inhibitor S3I-201 was obtained from Selleckchem (Houston, TX).

### Immunoprecipitation (IP), chromatin immunoprecipitation (ChIP), and ChIP/re-ChIP assays

To determine the SHOX2-STAT3 interaction, SHOX2 overexpressing T47D cell lysates (2 mg) were immunoprecipitated using anti-FLAG antibody and immunoblotted with anti-SHOX2 and anti-STAT3 antibodies. IgG was used as a negative control. ChIP assays were performed using a ChIP assay kit (MilliporeSigma, Burlington, MA) as described previously [16, 17]. The SHOX2 antibody used for the ChIP assay was obtained from Abcam (Cambridge, MA). For re-ChIP assays, complexes from the initial ChIP using anti-SHOX2 antibody were eluted with 100 µl of 10 mM DTT at 37°C for 30 min and diluted 1:20 in IP dilution buffer. The eluates were re-immunoprecipitated with a control IgG or anti-STAT3 antibody. Each immunoprecipitated DNA sample was quantified by qPCR using the primers (F: 5’-ATTAATGGCCAAGAGCACAG-3’; R: 5’-CGGCCTCTTGTTTTGAGTAA-3’) that were designed to amplify a proximal promoter region containing putative SHOX2 binding sites on the WASF3 promoter. All samples were run in triplicate, and results were averaged after normalization to the input.

### DNA methylation analysis

Genomic DNA was purified using the DNeasy Tissue Kit (Qiagen, Valencia, CA) and sodium bisulfite treatment of genomic DNA was carried out using the DNA Modification Kit (Zymo Research, Irvine, CA). For DNA sequencing, the bisulfite-modified genomic DNA was used as the template for PCR amplification of the WASF3 promoter region. Bisulfite sequencing PCR primers (F: 5’- AGGTAGAGGATTTTTGTGTAGGAG-3’; R: 5’- AAAATAAACRCRAAAAACTACA-3’) used to amplify the region of the WASF3 promoter were designed using the MethPrimer program. The amplified DNA fragments were cloned into pCR2.1 vector (Invitrogen), and individual clones were sequenced.

### Animal studies

All experimental procedures were approved by the Institutional Animal Care and Use Committee (IACUC) of Georgia Regents University. Six-week-old female NOD.Cg-*Prkdc*^*scid*^*Il2rg*^*tm1Wjl*^*/*SzJ (NSG) mice were purchased from the Jackson Laboratory (Bar Harbor, ME, USA) and maintained in accordance with IACUC guidelines. The orthotopic xenograft mice were established by injection of MDA-MB-231 cells into the second mammary fad pat at the base of the nipple. Tumor growth was measured externally every 4 to 7 days using vernier calipers and the tumor volume was calculated as length × width^2^ × 0.52. The mice were euthanized at the endpoint and the lungs were removed from these mice for histological analyses as described previously [18]. Metastatic nodules in mouse lungs was determined by counting the number of metastatic foci on the lung surface. The metastasis index was defined as the percentage of total metastatic nodule area to the total lung area based on the calculation from 15 slides.

### Immunohistochemistry (IHC)

Sections of breast xenograft tumors were immunostained with anti-Ki67 (1:2000, Abcam) or E-cadherin antibody (1:500, Abcam) as described previously [14, 15]. Negative controls included non-specific polyclonal rabbit antibody at 2 µg/ml (Abcam, Cambridge, MA). The sections were developed with the diaminobenzidine tetrahydrochloride (DAB) substrate kit (Vector Laboratories) and counterstained with hematoxylin.

### Bioinformatics and statistical analysis

The gene expression data and corresponding clinicopathological data for 1099 breast cancer patients were extracted from The Cancer Genome Atlas (TCGA, https://cancergenome.nih.gov/) using either the UCSC Xena platform or cBioPortal [19]. To determine the influence of SHOX2 expression on progression-free and disease-specific survival of breast cancer patients, the upper and lower quartiles of patients were stratified based on high and low SHOX2 mRNA expression and a Kaplan-Meier survival analysis was performed. A comparison of various treatment effects was performed using either one-way analysis of variance (ANOVA), a two-tailed Student’s *t*-test, or a Pearson correlation (GraphPad Prism 9). Statistical examination of in vivo animal data utilized Fisher analysis among the different treatment groups. Differences with a *p*-value of < 0.05 were considered statistically significant. Experiments shown are the means of multiple individual points from multiple experiments (± S.D.).

## Results

### SHOX2 expression corresponds with breast cancer patient severity

**Fig. 1:**
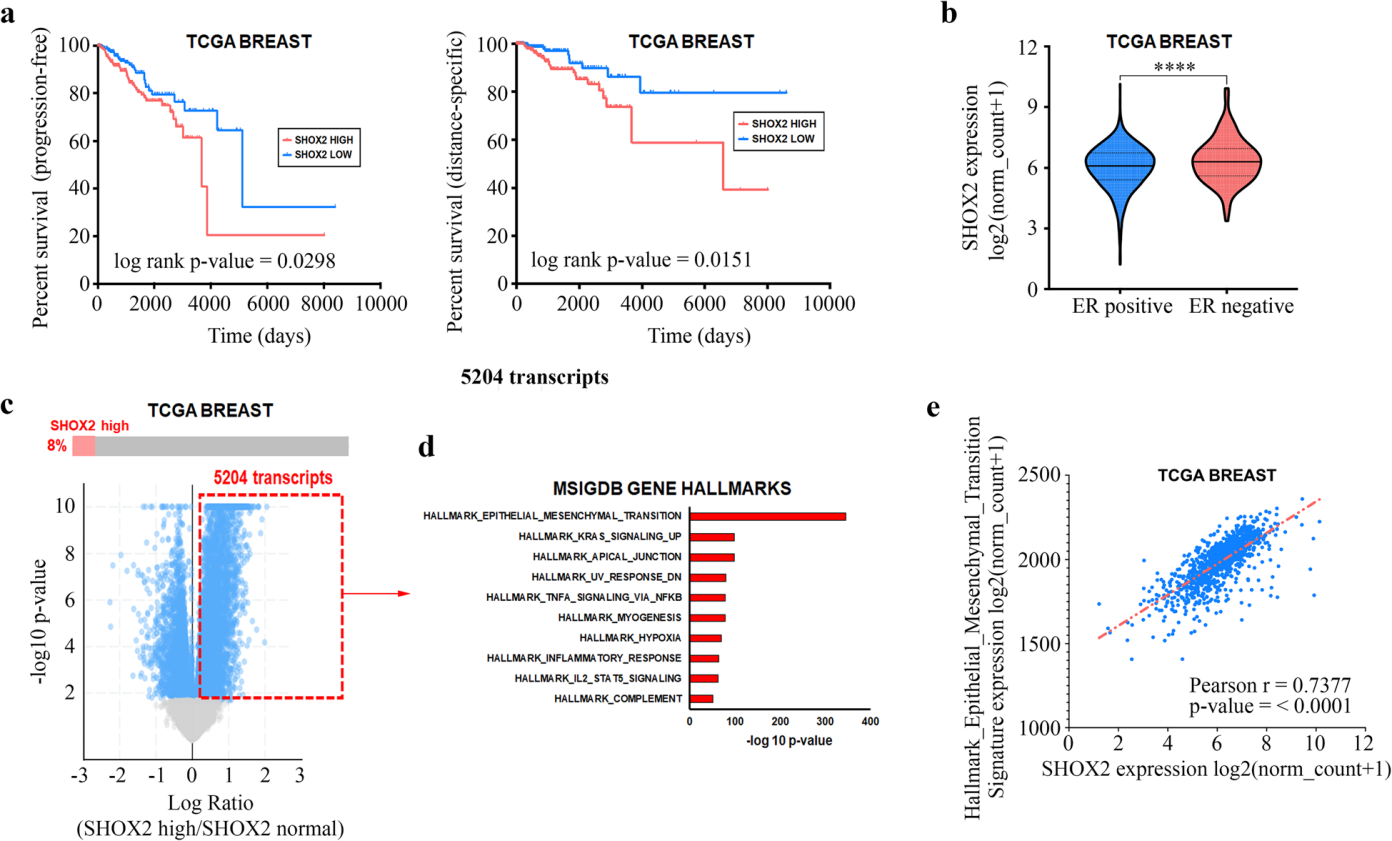
SHOX2 in TCGA breast cancer patient samples. (**a**) Higher SHOX2 expression corresponds with significantly worse progression free survival and disease-specific survival. (**b**) SHOX2 expression is differentially overexpressed in estrogen receptor-negative (ER^−^) breast tumors when compared to estrogen receptor-positive (ER^+^) tumors. **** *p* < 0.0001. (**c, d**) Breast tumors deemed “SHOX2-high” tumors (z-score > 1.25) differentially overexpressed transcripts preferentially involved in the epithelial-to-mesenchymal transitioning. (**e**) SHOX2 expression significantly correlates with the MSigDB Gene Hallmark dataset “EPITHELIAL_MESENCHYMAL_TRANSITION”

To gain initial insight into the role of SHOX2 expression in breast cancer, we analyzed TCGA breast cancer cohort to determine how SHOX2 expression corresponded with specific clinical characteristics. We first determined that high SHOX2 expression corresponded with both worse progression-free survival as well as disease-specific survival when comparing the highest quartile of patient SHOX2 expression (n = 272 and 268, respectively) with the lowest quartile of patients SHOX2 expression (n = 272 and 260, respectively) (Fig. 1a). Moreover, the microarray and survival data from GSE2034 support the correlation between higher SHOX2 expression and worse relapse-free survival (Supplementary Fig. S1). This worse prognostic feature of high SHOX2 expression also corresponded with ER negative (ER^−^) breast tumors having overall higher SHOX2 expression than ER positive (ER^+^) breast tumors (*p* < 0.0001) (Fig. 1b). However, we did not observe any noticeable changes in SHOX2 expression between four subtypes, especially when considering the basal-like subtype and its relationship with triple negative status (Supplementary Fig. S2).

We next determined the differentially gene expression profile of TCGA breast patient tumors demonstrating high SHOX2 expression (z-score > 1.25) and observed a significant enrichment of transcripts associated with the Molecular Signatures Database (MsigDB) Gene Hallmark dataset EPITHELIAL_MESENCYMAL_TRANSITION represented in this subset of samples (Fig. 1c and d). Furthermore, this gene expression signature of Gene Hallmark EPITHELIAL_MESENCYMAL_TRANSITION significantly correlated with SHOX2 expression (r = 0.7377) in TCGA breast cancer patients (Fig. 1e). Based on these results, we found a clinical connection between SHOX2 expression and aggressive, metastatic characteristics in breast cancer patients.

### SHOX2 contributes to breast tumor growth and metastasis

**Fig. 2:**
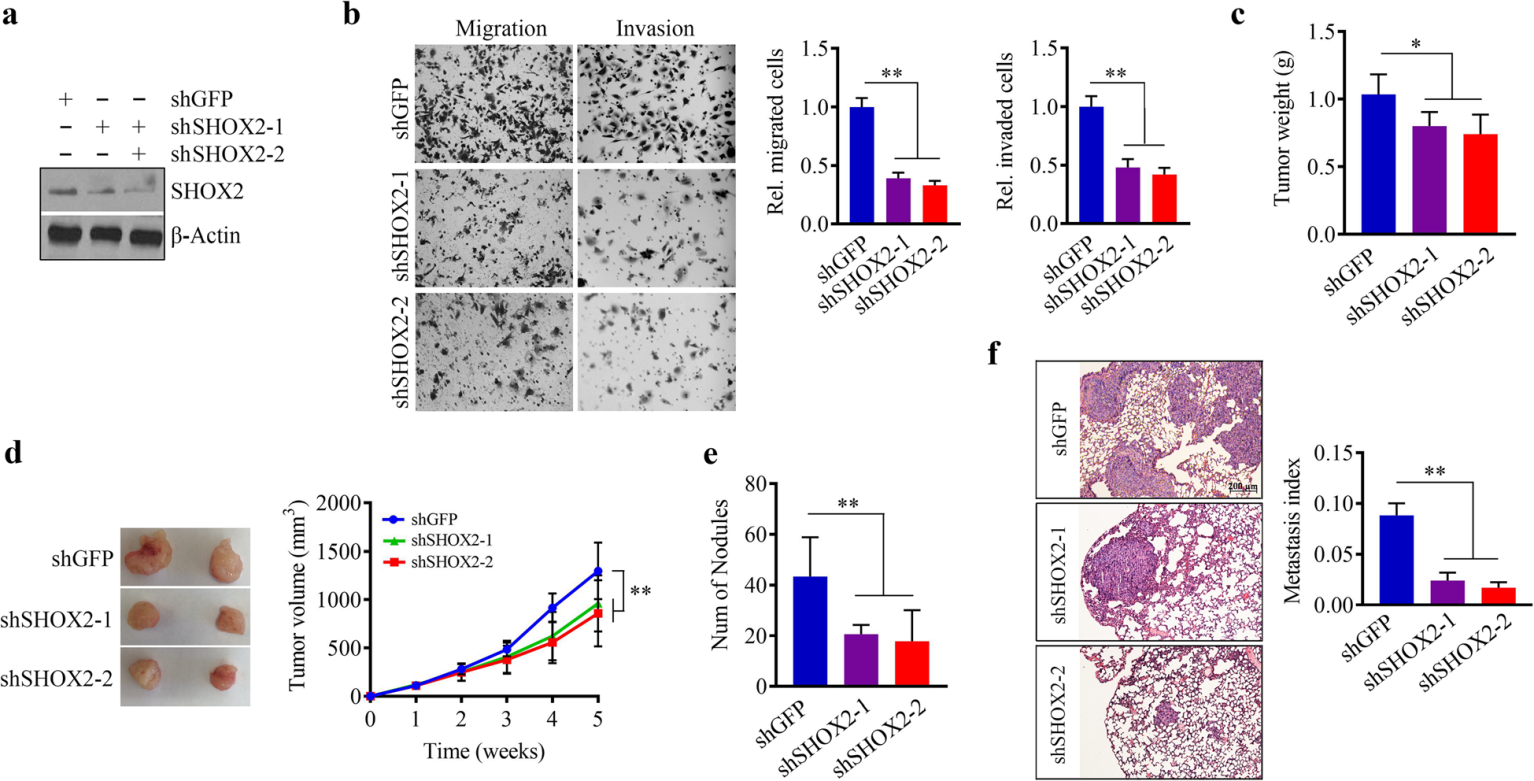
SHOX2 plays an important role in breast cancer metastasis. (**a**) The knockdown effect of specific shRNAs on SHOX2 expression in MDA-MB-231 cells. (**b**) The effect of SHOX2 knockdown on migration and invasion of MDA-MB-231 cells. Representative images and quantitative data are shown in the left and right panels, respectively. **(c, d**) The effect of SHOX2 knockdown on breast tumor growth in MDA-MB-231-bearing NSG mice (n = 5 mice/group). Tumor weight and growth curves are shown in (**c**) and (**d**), respectively. (**e, f**) The effect of SHOX2 knockdown on breast tumor lung metastasis in MDA-MB-231-bearing NSG mice. The number of nodules on the lung surface and metastasis index measured on H&E-stained lung sections are shown in (**e**) and (**f**), respectively. shGFP: the control shRNA against GFP; shSHOX2-1 and shSHOX2-2: two shRNAs targeting different sequences of SHOX2. ^*^p < 0.05, ^**^p < 0.01

SHOX2 are highly expressed in mesenchymal-like breast cancer cell lines while are either low or undetectable in epithelial-like breast cancer cell lines [8]. Base on TCGA results, we applied lentivirus-mediated shRNAs to deplete SHOX2 expression in ER^−^ MDA-MB-231 cells (Fig. 2a) which exhibit high metastatic and mesenchymal features as well as express high levels of SHOX2 when compared to ER^+^ T47D cells. Consistent with the previous report [8], SHOX2 knockdown remarkably reduced the cell potential in migration and invasion (Fig. 2b). To investigate the importance of SHOX2 expression in the tumorigenesis and metastasis of human breast cancer, we established orthotopic breast cancer xenografts by injection of SHOX2 knockdown and knockdown control MDA-MB-231 cells into the mammary fat pad of female NSG mice, respectively. In mice implanted with SHOX2 knockdown cells, tumors were significantly smaller, as measured by tumor weight and volume, compared with mice that were implanted with knockdown control cells (Fig. 2c and d). On the lung surface, a large number of metastatic lung lesions were observed in mice receiving knockdown control cells, while this number was greatly reduced in the mice implanted with SHOX2 deficient MDA-MB-231 cells (Fig. 2e). Specifically, these observations were confirmed by microscopic examination of lung tissue sections, which showed sharply decreased metastasis index in the lung (Fig. 2f). These findings indicate that loss of SHOX2 is sufficient to decrease the tumor growth and metastatic capacities of breast cancer cells.

### SHOX2 transcriptionally activates WASF3 expression in breast cancer cells

**Fig. 3:**
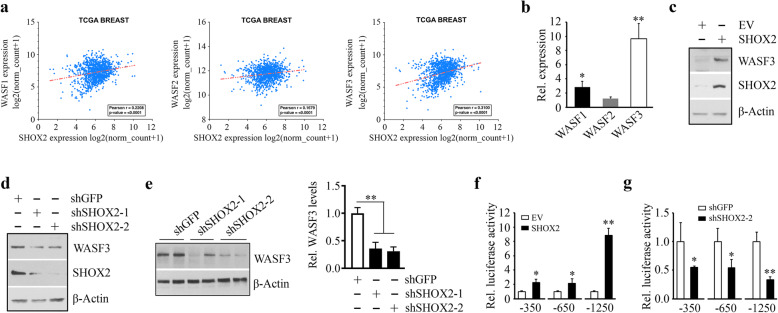
SHOX2 directly activates WASF3 gene transcription. (**a**) The positive correlation between the expression of SHOX2 and three WASF genes illustrated in scatter plot from the TCGA breast cancer cohort. (**b**) The effect of SHOX2 overexpression on WASF gene expression in T47D cells determined by QRT-PCR. (**c, d**) The effect of SHOX2 gene manipulations on WASF3 expression in T47D (**c**) and MDA-MB-231 (**d**) cells determined by Western blot. (**e**) The protein levels of WASF3 in xenograft breast tumors derived from SHOX2 knockdown and the knockdown control MDA-MB-231 cell. Representative images and quantitative data (*n* = 5 mice/group) are shown in the left and right panels, respectively. (**f, g**) The effect of SHOX2 gene manipulations on the luciferase activity in T47D (**f**) and MDA-MB-231 (**g**) cells transfected with the constructs harboring different lengths of the WASF3 promoters. ^*^p < 0.05, ^**^p < 0.01

To elucidate the mechanisms underlying SHOX2-mediated metastasis, we examined the correlation between the expression of SHOX2 and metastatic drivers using the gene expression data from the TCGA breast cancer cohort (Fig. 3a). WASF members appear to play a major role not only in the regulation of actin cytoskeleton dynamics but also in cancer cell invasion/metastasis [18, 20, 21]. In this analysis, SHOX2 expression levels were found to be significantly correlated with the expression of two WASF genes, WASF1 and WASF3 (Fig. 3a), suggesting SHOX2 may promote breast cancer metastasis through WASF-mediated signaling.

SHOX2 is a homeodomain transcription factor containing a 60-amino acid residue motif representing a DNA-binding domain [8]. Interestingly, analysis of the WASF gene promoters for HOX-binding consensus sequence (ATTA(N)_n_TAAT) showed three, one, and four putative SHOX2 binding sites within the promoter of WASF1, WASF2, and WASF3 genes, respectively (Supplementary Fig. S3). To identify the functional correlation between SHOX2 and WASF proteins, we conducted QRT-PCR analysis with T47D cells overexpressing SHOX2 or an empty vector. Consistent with TCGA data, SHOX2 overexpression led to an increase in both WASF1 and WASF3 genes but not WASF2 (Fig. 3b). Compared with WASF1, the expression levels of WASF3 increased more significantly (approximately 10-fold greater in T47D cells) when SHOX2 was overexpressed (Fig. 3b). Western blot analysis further showed that overexpression of SHOX2 increased WASF3 protein levels in T47D cells (Fig. 3c). In contrast, reduced WASF3 levels were seen in SHOX2 knockdown MDA-MB-231 cells (Fig. 3d) and the xenograft tumors derived from SHOX2 knockdown MDA-MB-231 cells (Fig. 3e). These findings support the notion that SHOX2 positively regulates WASF3 expression in breast cancer cells.

Given that our previous studies have demonstrated WASF3 to be the most closely associated with breast cancer invasion and metastasis of the WASF family members [18, 20, 21], we subsequently focused our attention on the regulation of WASF3 by SHOX2. As part of our ongoing characterization of the WASF3 promoter, three WASF3 promoter constructs (-350/+494, -650/+494, and − 1250/+494) were generated from sequences upstream the WASF3 transcription start site (TSS), which contained one, two and four putative SHOX2 binding sites, respectively. To identify which DNA regulatory element within the WASF3 gene was essential for transactivation by SHOX2, we transfected these different lengths of WASF3 promoter constructs into SHOX2 overexpressing T47D cells and determined the luciferase activities. As shown in Fig. 3f, SHOX2 overexpression resulted in a 9-fold increase in luciferase expression in the presence of the − 1250/+494 WASF3 reporter construct compared to that of the control cells. Significant activation of the luciferase reporters was also observed when SHOX2 expression was forced in the presence of the other two shorter reporter constructs (Fig. 3f). In contrast, knockdown of SHOX2 in MDA-MB-231 cells reduced the luciferase activities of the three reporter constructs (Fig. 3 g). Collectively, these data suggest that the putative SHOX2 binding sites located 1250 bp upstream of TSS within the promoter of the WASF3 gene (Supplementary Fig. S3) contribute to SHOX2-induced WASF3 transcriptional activation.

### SHOX2 and STAT3 cooperate to promote WASF3 transcriptional activation

**Fig. 4:**
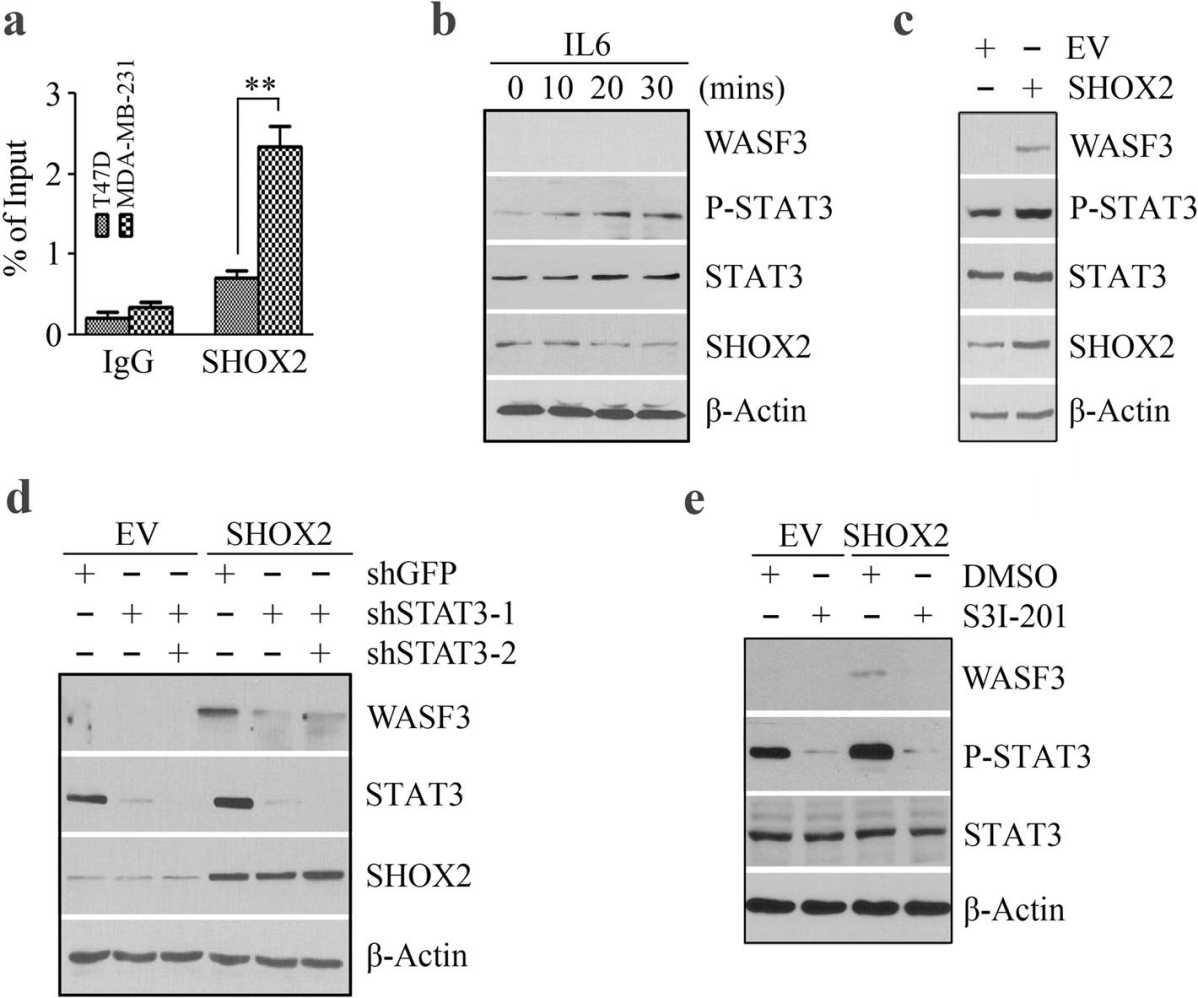
STAT3 cooperates with SHOX2 to promote WASF3 gene transcription. (**a**) The accumulation of SHOX2 on the WASF3 promoter was determined by ChIP-QPCR assays in MDA-MB-231 and T47D cells. (**b**) The effect of 20 ng/ml IL6 on the expression of WASF3 and SHOX2 in T47D cells. (**c**) The effect of SHOX2 overexpression on WASF3 and STAT3 expression and phosphorylation in T47D cells. (**d**) The effect of STAT3 knockdown (shSTAT3-1 and shSTAT3-2) on WASF3 expression in SHOX2 overexpressing T47D cells. (**e**) The effect of 50 µM S3I-201 on WASF3 expression in SHOX2 overexpressing T47D cells. ^*^p < 0.05, ^**^p < 0.01

To investigate whether the specific binding of SHOX2 to the WASF3 promoter was required for the transcriptional activation of WASF3, we carried out ChIP assays which demonstrated the specific occupancy of SHOX2 at the WASF3 promoter-binding sites in breast cancer cells (Fig. 4a). T47D cells did not express WASF3 (Figs. 3c and 4b), and the binding of SHOX2 in these cells was significantly weaker in comparison with MDA-MB-231 cells (Fig. 4a). These data indicate a strong association of SHOX2 occupancy with WASF3 expression.

Previously, we revealed that another transcription factor, STAT3, can be recruited to its DNA-binding elements within the WASF3 promoter to active WASF3 transcription [16, 21]. STAT3 is activated by tyrosine phosphorylation in response to a variety of cytokines, such as IL6, and subsequently translocates into the nucleus where it acts to regulate its downstream targets [10]. Increased phosphorylation levels of STAT3 were seen in WASF3 non-expressing T47D cells upon IL6 stimulation, but this increase did not upregulate either WASF3 or SHOX2 expression levels (Fig. 4b), suggesting that other factors may contribute to STAT3-mediated WASF3 transcriptional activation. Interestingly, a concordant increase in WASF3 expression and STAT3 phosphorylation were seen in T47D cells when SHOX2 was overexpressed (Fig. 4c), prompting us to examine the possibility that STAT3 cooperated with SHOX2 to regulate WASF3 transcriptional activation. To follow up, we depleted STAT3 expression in SHOX2 overexpressing and control T47D cells and evaluated the expression of WASF3. Overexpressing SHOX2 in cells that maintained STAT3 expression exhibited increased WASF3 protein levels (Fig. 4d), but these levels were dramatically attenuated following STAT3 knockdown, which did not directly affect the levels of SHOX2 (Fig. 4d). Inactivation of STAT3 with its small-molecule inhibitor S3I-201 also produced the same effect (Fig. 4e). These observations support the notion that STAT3 cooperates with SHOX2 to promote WASF3 transcriptional activation in breast cancer cells, or at least in T47D cells.

### SHOX2/STAT3 complex is assembled on the WASF3 promoter to activate its transcription

**Fig. 5:**
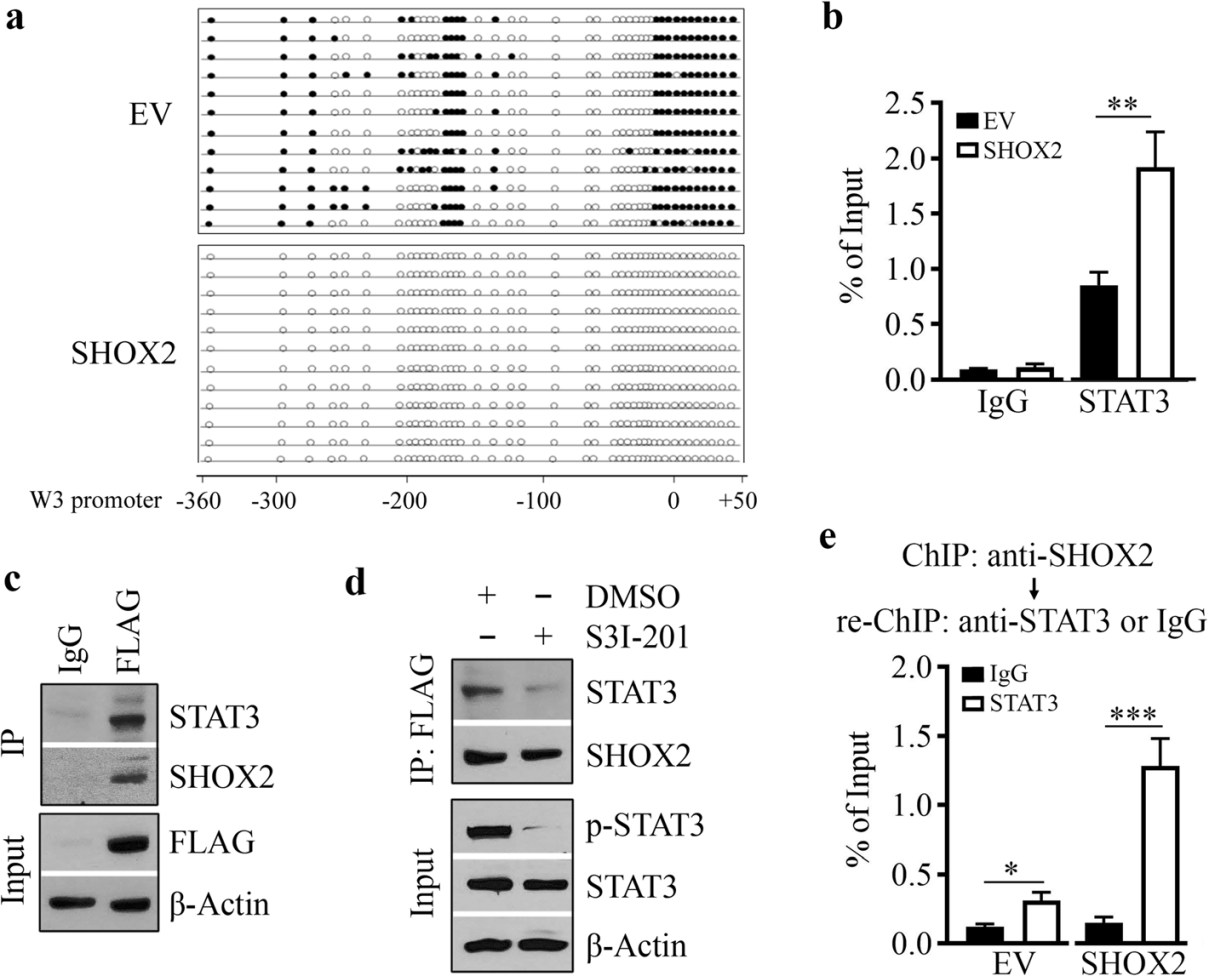
SHOX2 and STAT3 are assembled in the same complex on the WASF3 promoter. (**a**) CpG methylation level of the WASF3 promoter in SHOX2 overexpressing (SHOX2) and control (EV) T47D cells. Individual clones (n = 12) for each cell line were sequenced, and the methylation level of CpGs was analyzed based on the sequencing data (open circle, unmethylated CpG; closed circle, methylated CpG). (**b**) The accumulation of STAT3 on the WASF3 promoter was determined by ChIP-QPCR assays in SHOX2 overexpressing and control T47D cells. (**c**) The binding of SHOX2 to STAT3 was determined by IP assays in SHOX2 overexpressing T47D cells. (**d**) The effect of 50 µM S3I-201 on SHOX2-STAT3 interaction in overexpressing T47D cells. In (c) and (d), cell lysates were IP using an anti-FLAG antibody and immunoblotted with anti-STAT3 and anti-SHOX2 antibodies. IgG was served as a negative control. (**e**) The binding of the SHOX2/STAT3 complex on the WASF3 promoter was determined by ChIP and re-ChIP assays in SHOX2 overexpressing and its control T47D cells. Chromatin fragments immunoprecipitated with anti-SHOX2 antibody in the first ChIP were used as an input and were subjected to re-ChIP analysis using control IgG or anti-STAT3 antibody. ^*^p < 0.05, ^***^p < 0.001

Interestingly, DNA sequence analysis showed a high density of CpG sites in the WASF3 promoter region (Supplementary Fig. S4). To determine whether demethylation is one of the mechanisms contributing to SHOX2-mediated WASF3 transcriptional activation, methylation-sensitive PCR and DNA sequencing were used to measure the methylation level of the WASF3 promoter in SHOX2 overexpressing and control T47D cells. As shown in Fig. 5a, the majority of CpGs in the WASF3 promoter region of -360/+50 were methylated in T47D cells. In contrast, these CpGs were demethylated when T47D cells expressing SHOX2 (Fig. 5a), suggesting SHOX2-mediated demethylation facilitates its induced WASF3 upregulation. These results prompted a hypothesis that SHOX2 may facilitate STAT3 recruitment to the WASF3 promoter. To answer this, we performed ChIP assay with an anti-STAT3 antibody and found a higher amount of STAT3 was accumulated in the WASF3 promoter of T47D cells when SHOX2 was overexpressed (Fig. 5b). These observations thus indicate that promoting STAT3 to the methylated WASF3 promoter DNA is another mechanism involved in SHOX2-induced WASF3 upregulation in breast cancer cells.

To determine whether STAT3 was one of the SHOX2-interacting protein partners, we immunoprecipitated SHOX2 protein with anti-FLAG antibody in SHOX2 overexpressing T47D cells and immunoblotted with anti-STAT3 antibody. This analysis showed that STAT3 was present in the SHOX2 immunocomplex (Fig. 5c). We then examined the importance of STAT3 phosphor-activation in the STAT3-SHOX2 interaction by measuring STAT3 levels in the SHOX2 immunocomplex in SHOX2 overexpressing T47D cells in the presence or absence of S3I-201. IP results showed that S3I-201 remarkably blocked the association of STAT3 with SHOX2 (Fig. 5d), suggesting that STAT3 phosphor-activation is required for SHOX2/STAT3 immunocomplex formation. To test the possibility that SHOX2 and STAT3 were assembled on the WASF3 promoter, we performed ChIP/re-ChIP assays. Intriguingly, the STAT3 re-ChIP assay with anti-SHOX2 immunoprecipitates led to the precipitation of the WASF3 promoter DNA fragment (Fig. 5e), indicating that STAT3 and SHOX2 were bound together on the same WASF3 promoter. These results demonstrate the coexistence of STAT3 and SHOX2 on the WASF3 promoter, which is essential for promoting WASF3 transcriptional activation in breast cancer cells.

### WASF3 is required for SHOX2-dependent cell invasion and EMT

**Fig. 6:**
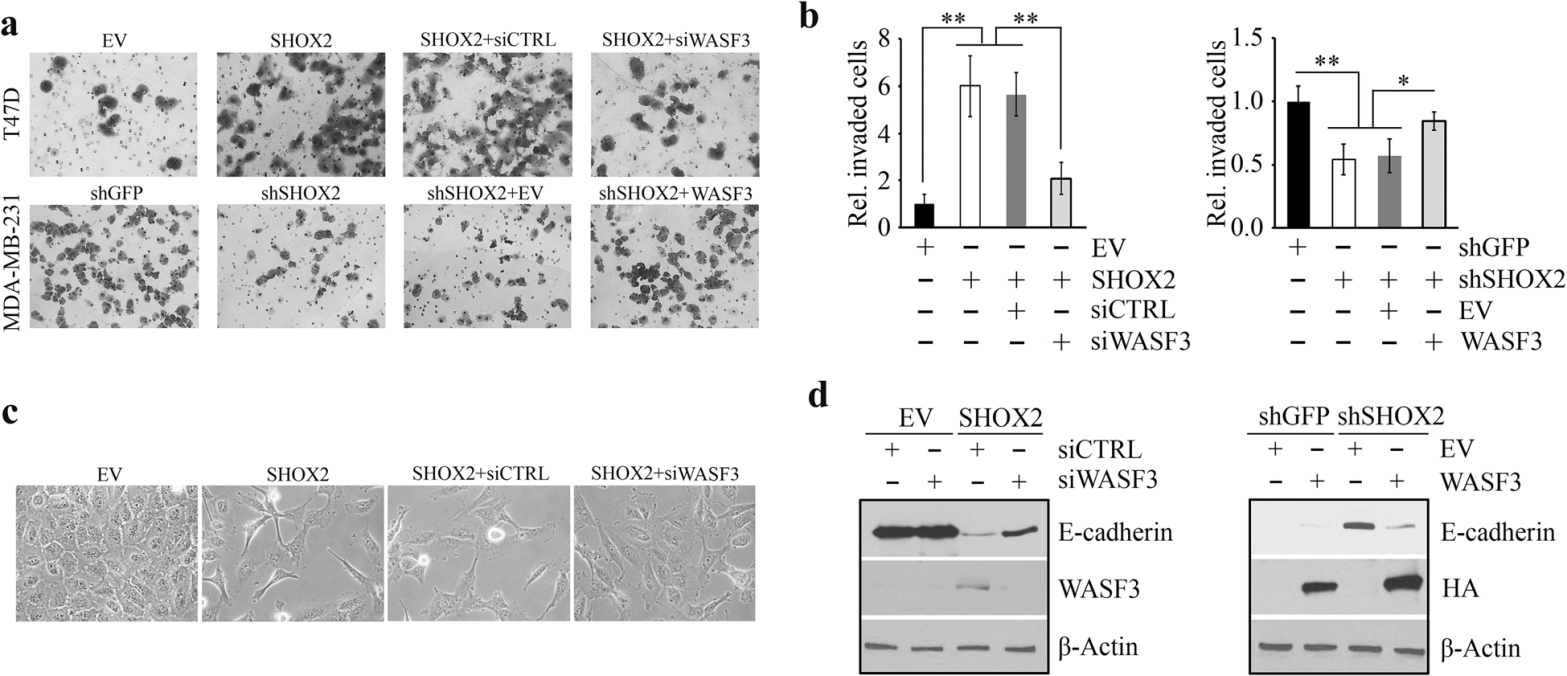
WASF3 is critical for SHOX2-mediated breast cancer cell invasion. (**a, b**) The effect of WASF3 gene manipulations on cell invasion in SHOX2 overexpressing T47D cells and knockdown MDA-MB-231 cells. Quantitative data of (**a**) are shown in (**b**). (**c**) The effect of WASF3 knockdown on EMT induced by SHOX2 overexpression in T47D cells. (**d**) The effect of WASF3 gene manipulations on E-cadherin levels in SHOX2 overexpressing T47D cells and SHOX2 knockdown MDA-MB-231 cells. ^*^p < 0.05, ^**^p < 0.01

The transcriptional regulation of WASF3 by SHOX2 encouraged us to determine whether WASF3 was a functional downstream target of SHOX2 during its promotion of breast cancer metastasis. We subsequently depleted WASF3 by siRNA in SHOX2 overexpressing T47D cells and found that loss of WASF3 expression significantly attenuated SHOX2-dependent cell invasion (Fig. 6a and b). Consistent with this, a loss of invasion was also seen in SHOX2 knockdown MDA-MB-231 cells, which could be partially rescued by restoring WASF3 expression (Fig. 6a and b). However, no significant changes in proliferation were observed when WASF3 expression levels were modulated in these cells (Supplementary Fig. S5). Notably, overexpression of SHOX2 facilitated the transition of epithelial T47D cells to a mesenchymal phenotype (Fig. 6c) associated with decreased E-cadherin levels (Fig. 6d), indicating that SHOX2 regulates cell invasion by promoting the EMT process. Depleting WASF3 in these cells antagonized SHOX2-regulated EMT (Fig. 6c) and the suppression of E-cadherin (Fig. 6d). Conversely, restoring WASF3 in MDA-MB-231 cells prevented the increase of E-cadherin levels resulting from SHOX2 depletion (Fig. 6d). These results signify that WASF3 plays an essential role in SHOX2-induced invasion and EMT in breast cancer cells.

### The SHOX2-WASF3 signaling is critical for breast cancer metastasis

**Fig. 7:**
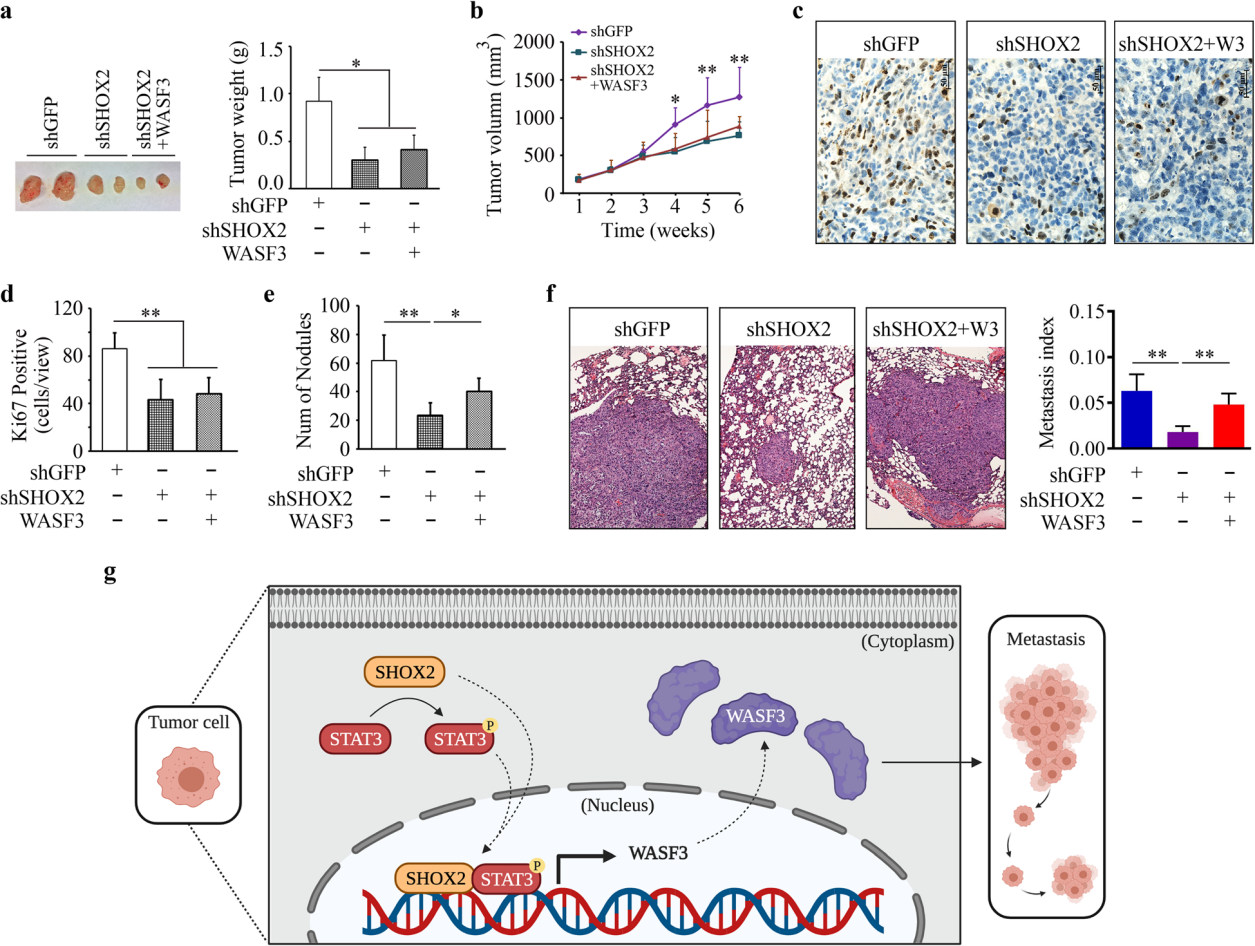
SHOX2 drives breast cancer metastasis in a WASF3-dependent manner. (**a-d**) The effect of WASF3 overexpression on tumor growth repressed by SHOX2 knockdown in MDA-MB-231-bearing NSG mice (n = 5/group). Tumor weight, growth curves, representative Ki67 immunostaining, and quantitative data of Ki67-positive cells are shown in (**a**), (**b**), (**c**), and (**d**), respectively. (**e, f**) The effect of WASF3 overexpression on lung metastasis repressed by SHOX2 knockdown in MDA-MB-231-bearing NSG mice. The number of nodules on the lung surface and metastasis index measured on H&E-stained lung sections are shown in (**e**) and (**f**), respectively. (**g**) Proposed model for the transcriptional regulation of WASF3 by SHOX2/STAT3 signaling in breast cancer cells. ^*^p < 0.05, ^**^p < 0.01

We next investigated whether the SHOX2-WASF3 signaling was involved in breast cancer metastasis using an NSG orthotopic xenograft model. In line with the findings shown in Fig. 2, SHOX2 knockdown MDA-MB-231 cells formed smaller primary tumors in the mammary fat pad (Fig. 7a and b) with reduced enrichment of Ki67-positive cells in the xenograft tumors (Fig. 7c and d) when compared with the knockdown control group. Interestingly, restoration of WASF3 expression in MDA-MB-231 cells did not alleviate the suppressive effect of SHOX2 loss on tumor outgrowth, as evidenced by similar primary tumor sizes (Fig. 7a and b) and Ki67-positive cancer cell numbers (Fig. 7c and d). The loss of pulmonary metastatic potential seen in the mice implanted with SHOX2 knockdown cells, however, was significantly increased when WASF3 expression was restored in these cells (Fig. 7e and f). In addition, increased levels of E-cadherin were consistently seen in xenograft tumor tissues derived from SHOX2 knockdown cells compared with the knockdown control cells, which were dramatically suppressed in xenograft tumors when WASF3 expression levels were restored in SHOX2 knockdown cells (Supplementary Fig. S6). Collectively, these findings strongly support the critical role of the SHOX2-WASF3-E-cadherin signaling in breast cancer metastasis.

## Discussion

As a crucial transcriptional regulator in several genetic disorders, SHOX2 has been demonstrated to be an excellent biomarker in the diagnosis and evaluation of many types of cancers, including lung cancer [22, 23]. SHOX2 expression also appears to be a functional prognostic marker and has been used as one of the independent indicators for grade II and III diffuse gliomas [24]. Besides this, elevated SHOX2 expression correlates with tumor recurrence in hepatocellular carcinoma [25]. In the present study, our TCGA data established the connection between SHOX2 abundance and a high gene expression signature of Gene Hallmark EPITHELIAL_MESENCYMAL_TRANSITION in breast cancer patients, providing a strong rationale for targeting SHOX2 in metastatic breast cancer. Previously, we probed the function of SHOX2 in cancer metastasis in zebrafish and found that loss of SHOX2 expression can inhibit the dissemination of MDA-MB-231 cells throughout the fish body [8]. Using orthotopic xenograft NSG mice, we now have further demonstrated that knockdown of SHOX2 in these cells reduces their risk of metastatic spread. Together, these results indicate the involvement of SHOX2 in breast cancer progression, likely due to its role in enhancing the proliferation and invasion potential of cancer cells.

STAT3 has been consistently implicated in promoting cancer cell invasion and metastasis in many cancer types and was shown here to bind to its cognate elements in the WASF3 promoter [10, 16]. We have established the STAT3-WASF3 metastatic signaling in breast cancer cells [16]. The levels of SHOX2 mRNA are shown much lower or undetectable in epithelial-like cell lines (such as T47D cells) compared with those in mesenchymal-like cell lines (such as MDA-MB-231 cells) [8]. Upregulating STAT3 expression or activating it in response to IL6 treatment leads to increased WASF3 expression as well as invasion potential in MDA-MB-231 cells [16], but this is not true in WASF3 non-expression T47D cells, implying that the abundance of SHOX2 is a critical factor in the STAT3-mediated regulation of WASF3. The data presented here provide the first evidence that the transcriptional activation of WASF3 is synergistically regulated by SHOX2 and STAT3 through their assembly of a functional complex on the WASF3 promoter in breast cancer cells (Fig. 7 g).

The reported upregulation of SHOX2 by STAT3 activation in *Helicobacter pylori*-infected gastric cancer cells [7] is a cell type-specific effect given that no elevation in SHOX2 was observed in breast cancer cells following IL6-induced STAT3 activation. In contrast, enforced expression of SHOX2 leads to an increase in phosphor-STAT3 levels in T47D cells, although the mechanistic explanation for this is currently lacking. It also remains to be determined how general this observation is in other cancer cell lines. Methylation was believed to play a pivotal role in repressing gene expression, perhaps by blocking the promoters at which activating transcription factors should bind. In this study, we show promoter hypermethylation of the WASF3 gene in T47D cells and demethylation of -360/+50 by SHOX2 facilitates WASF3 upregulation. Given that IL6 stimulation increases WASF3 expression in MDA-MB-231 cells [16] but not T47D cells, the possibility that binding of SHOX2 and STAT3 transcription factors precedes loss of methylation on the WASF3 promoter is excluded. It appears that SHOX2 facilitates WASF3 demethylation on CpG enrich region (the first level), which allows SHOX2 and STAT3 to bind to the WASF3 promoter to initiate its transcription (the second level). Nevertheless, our study underscores the complexity of WASF3 transcriptional activation and provides evidence that SHOX2 and STAT3 are assembled in the same complex on the WASF3 promoter and act synergistically to promote WASF3 expression in breast cancer cells. Further exploration of the mechanism of SHOX2 on DNA methylation is warranted.

WASF1 has been implicated in cell movement through its regulation of membrane structures [12, 26]. Our study shows that the manipulation of SHOX2 expression levels also affects the transcriptional activation of the WASF1 gene. We have demonstrated previously that, unlike WASF3, WASF1 is not required for the invasion and metastasis of breast cancer cells [20]. Therefore, although both WASF1 and WASF3 genes are SHOX2 targets and their transcript levels are positively regulated by SHOX2, the SHOX2-dependent promotion of metastasis appears to be due to its ability to influence the expression of WASF3. SHOX2 is reported to activate the TGF-β receptor I (TβR-I) gene through the consensus HOX-binding site in its promoter, leading to EMT induction and invasion through the TGF-β signaling network in epithelial-like breast cancer cells [8]. Here, we reveal that SHOX2 signaling additionally influences the metastatic process through WASF3/E-Cadherin signaling in breast cancer cells. When combined, these effects may further impact SHOX2’s control of metastasis.

Metastasis is the hallmark of cancer that is responsible for the most significant number of cancer-related deaths. Although vital advances have been made in detecting and combatting cancer, particularly at its early stages, metastasis remains a formidable and frequently fatal challenge [27–30]. Overall, however, our findings provide new mechanistic insight into SHOX2-dependent cell invasion and metastasis and hint that inhibiting it may represent a promising therapeutic strategy for breast cancer patients.

## Conclusions

The results of this study suggest that SHOX2 cooperates with STAT3 to drive breast cancer metastasis through upregulating the metastasis-promoting gene WASF3. This novel and significant finding improves our mechanistic understanding of the metastatic disease. Moreover, interrupting the SHOX2-STAT3-WASF3 signaling axis within the metastatic cascade may represent a promising anticancer strategy for therapeutic purposes, which has a potential impact on extending survival in patients with metastatic breast cancer.

## Supplementary Information

**Additional file 1:**

## Data Availability

All data generated or analyzed during this study are included in this published article [and its supplementary information files].

## Abbreviations

ChIP: Chromatin immunoprecipitation
ChIP-qPCR: Chromatin immunoprecipitation coupled with qPCR
DAB: Diaminobenzidine tetrahydrochloride
EMT: Epithelial-mesenchymal transition
H&E: Hematoxylin-and-eosin
IACUC: Institutional Animal Care and Use Committee
IHC: Immunohistochemstry
IP: Immunoprecipitation
MsigDB: Molecular Signatures Database
NSG: NOD.Cg-*Prkdcscid Il2rgtm1Wjl/SzJ*
qRT-PCR: Quantitative RT-PCR
shRNA: Short hairpin RNA
siRNA: Small interfering RNA
SHOX2: Short stature homeobox 2
STAT3: Signal transducer and activator of transcription 3
TCGA: The Cancer Genome Atlas
WASF3: Wiskott-Aldridge syndrome protein family member 3
TβR-I: TGF-β receptor I
