# Supplementary material for: SHOX2 cooperates with STAT3 to promote breast cancer metastasis through the transcriptional activation of WASF3
Source: J Exp Clin Cancer Res. 2021 Aug 31;40:274. doi: 10.1186/s13046-021-02083-6 (PMC8406721; doi:10.1186/s13046-021-02083-6)
Supplement: Supplementary file 1 — Additional file 1: [file 13046_2021_2083_MOESM1_ESM.docx]

**Supplemental Data for**

**SHOX2** **cooperates with STAT3 to promote breast cancer metastasis through the transcriptional activation of WASF3**

Yong Teng, Reid Loveless, Elayne M Benson, Li Sun, Austin Y Shull, Chloe Shay

**Correspondence to:** Yong Teng, [yong.teng@emory.edu](mailto:yong.teng@emory.edu)

**This file includes:**

**Supplementary Figures 1-6 and Figure legends**

**Supplementary Figures S1.** Relapse-free survival of SHOX2 expression in GSE2034.

**Supplementary Figures S2.** The expression of SHOX2 in breast cancer molecular subtypes from the TCGA breast cancer cohort.


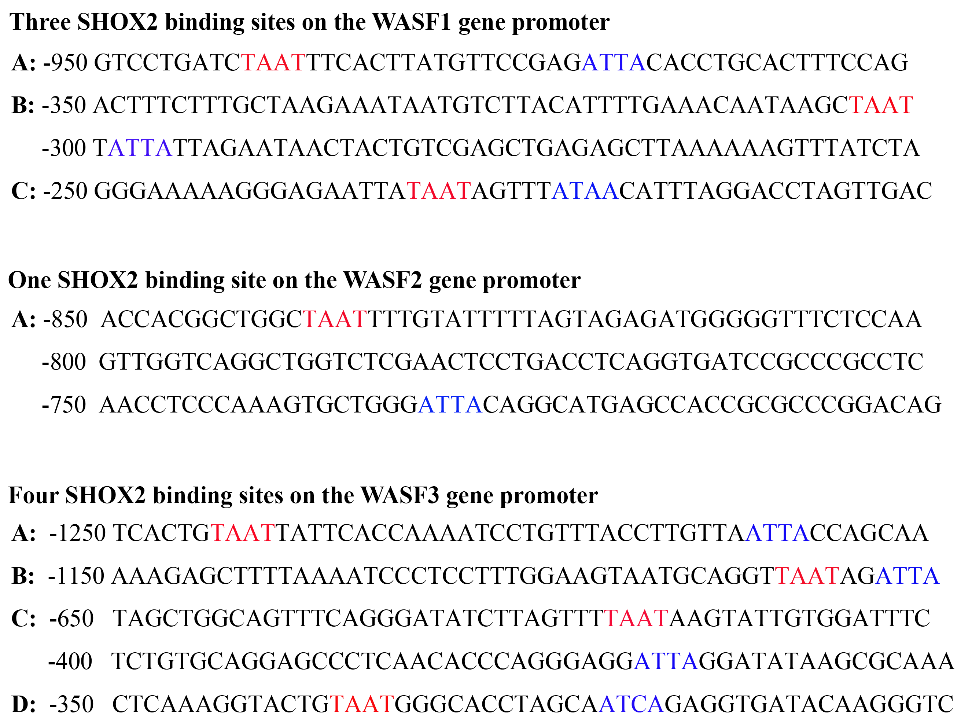


**Supplementary Figures S3.** The putative binding site(s) of SHOX2 (ATTA(N)nTAAT) on the promoters of WASF family genes.


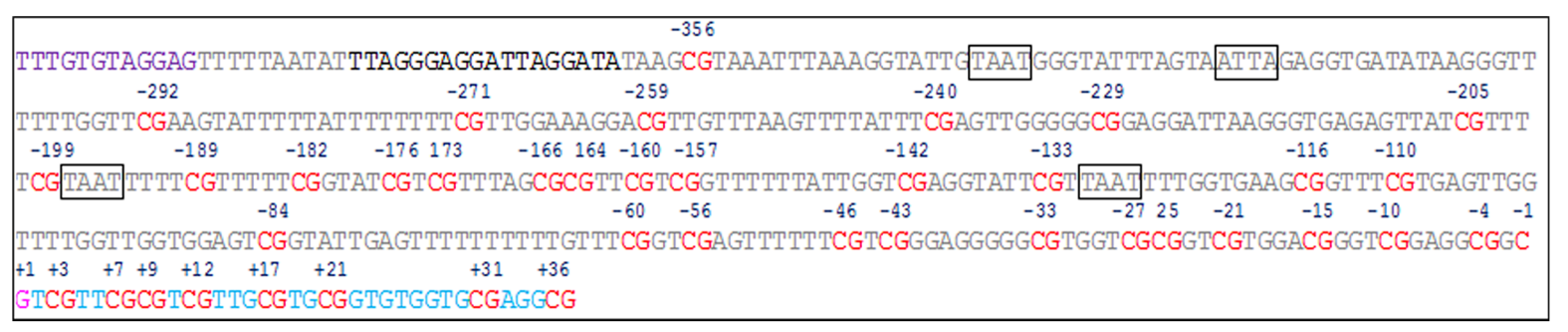


**Supplementary Figures S4.** Promoter analysis showing high-density CpG sites (red) and multiple SHOX2 binding sites (ATTA(N)nTAAT) (boxes) on the WASF3 gene promoter.


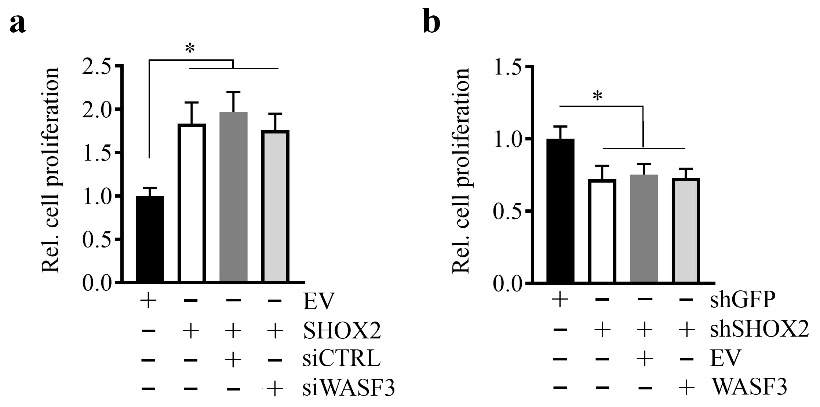


**Supplementary Figures S5.** Cell proliferation determined in SHOX2 overexpressing T47D cells with or without the WASF3 knockdown (a) and in SHOX2 knockdown MDA-MB-231 cells with or without WASF3 overexpression (b). siCTRL: a non-targeting siRNA; EV: an empty vector; ^*^ *p*<0.05.


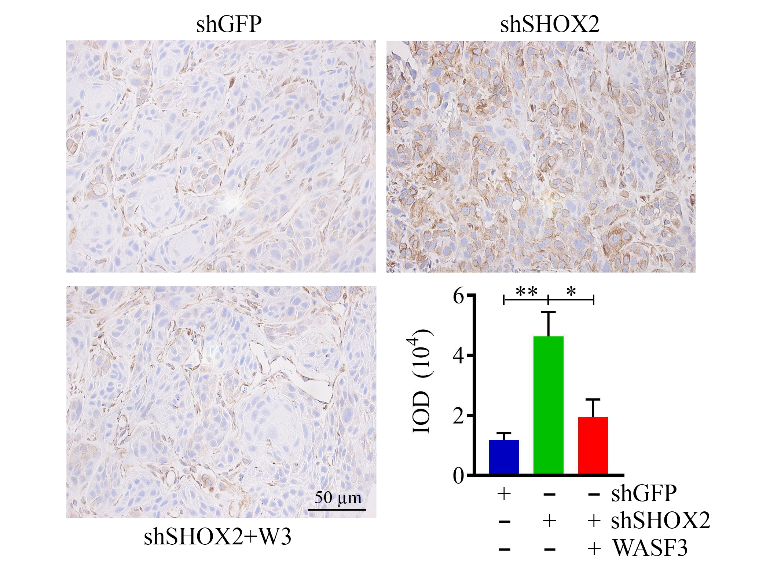


**Supplementary Figures S6.**  The protein levels of E-cadherin in xenograft tumors derived from the indicated gene-modified MDA-MB-231 cells determined by IHC. Representative E-cadherin immunostaining and quantitative data are shown. **p*<0.05; ***p*<0.01
